# Interocular Differences in Spatial Frequency Influence the Pulfrich Effect

**DOI:** 10.3390/vision4010020

**Published:** 2020-03-20

**Authors:** Seung Hyun Min, Alexandre Reynaud, Robert F. Hess

**Affiliations:** McGill Vision Research, Department of Ophthalmology and Visual Sciences, McGill University, Montreal, QC H3G 1A4, Canada; alexandre.reynaud@mail.mcgill.ca (A.R.); robert.hess@mcgill.ca (R.F.H.)

**Keywords:** interocular delay, Pulfrich effect, spatial frequency

## Abstract

The Pulfrich effect is a stereo-motion phenomenon. When the two eyes are presented with visual targets moving in fronto-parallel motion at different luminances or contrasts, the perception is of a target moving-in-depth. It is thought that this percept of motion-in-depth occurs because lower luminance or contrast delays the speed of visual processing. Spatial properties of an image such as spatial frequency and size have also been shown to influence the speed of visual processing. In this study, we use a paradigm to measure interocular delay based on the Pulfrich effect where a structure-from-motion defined cylinder, composed of Gabor elements displayed at different interocular phases, rotates in depth. This allows us to measure any relative interocular processing delay while independently manipulating the spatial frequency and size of the micro elements (i.e., Gabor patches). We show that interocular spatial frequency differences, but not interocular size differences of image features, produce interocular processing delays.

## 1. Introduction

**Figure 1:**
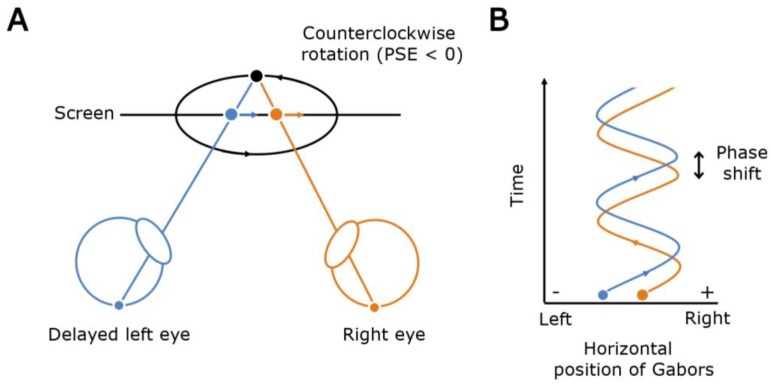
The Pulfrich effect as observed in our psychophysical task. (**A**) An illustration of the Pulfrich effect. When the left eye is delayed, the target moving rightward is seen behind its right eye image (blue and orange dots, respectively) resulting in an illusory uncrossed disparity (black dot). Hence, the cylinder in the task appears to be rotating in the counterclockwise direction. (**B**) A schematic showing how the presence of a phase shift (time, ordinate axis) between the horizontally moving Gabor elements shown to the right and left eyes induces a phase shift (spatial disparity) of the target image between the eyes (position, abscissa axis).

First reported nearly century ago, the Pulfrich effect is a visual illusion in which a horizontally moving stimulus such as a pendulum appears to follow an elliptical path in depth [1]. It occurs when one eye is delayed relative to the other eye (Figure 1A). Interocular delay can be caused by interocular differences in either luminance or contrast [2,3,4,5,6]. Hence, placing a neutral density filter over one eye produces an interocular luminance difference, delays the processing of said eye, and induces the Pulfrich effect.

According to the Fourier theory, a natural scene can be decomposed into a set of sine-wave gratings of different spatial frequencies, orientations, contrasts and phases [7]. High spatial frequency components subserve fine details of a visual scene (i.e., sharp edges and surface textures), whereas low spatial frequency components subserve coarse-scale information (i.e., overall shape and structures of objects). The visual system has a reduced sensitivity for high spatial frequencies owing to a combination of optical and neural imperfections [8]. Moreover, the processing speeds for fine and coarse scales (high and low spatial frequency respectively) also differ. Previous behavioral [9,10,11,12] and neurophysiological studies [13,14,15] have suggested that information at fine scales is processed more slowly than that at coarse scales. In addition, reaction times have been shown to increase when finer scale information within an image is processed [9,16]. Although the delay associated with the processing of finer scale image information (i.e., high spatial frequencies) could be partly due to the higher contrast thresholds associated with the detection of high spatial frequencies, a delay has nevertheless been observed for high-contrast, high-spatial-frequency stimuli [13]. Another metric of the speed of visual processing has been obtained from the visual evoked potential (VEP). Many electrophysiological studies have demonstrated that spatial frequency influences the latency of VEPs. For example, the latency of gratings of a higher spatial frequency, but a comparable contrast, is increased [17,18,19,20,21]. In short, the speed of visual processing for low-spatial-frequency information is faster than that for its high-spatial-frequency counterpart. 

Besides spatial frequency, size is another relevant spatial property of features within an image. Previous studies have reported that reaction times decrease as the area or size of a visual stimulus increases [22,23]. However, scaled-up stimuli of larger size contain lower spatial frequencies; therefore, the sole importance of size may have been confounded. 

In this study we investigate independently how spatial frequency and size of stimuli influence interocular delay using a novel psychophysical task based on the Pulfrich effect [5]. Reynaud and Hess (2017) introduced this novel task, in which dichoptically presented stimuli consisting of bandpass microelements differ in one stimulus parameter, such as contrast or luminance [5]. The task enables one to individually analyze the influence of a stimulus parameter on the interocular delay. In our study, we varied separately the spatial frequency and size (or bandwidth) of 200 Gabor elements, which were dichoptically displayed and defined a cylinder stimulus. A Gabor element is a product of a sinusoidal and Gaussian functions. On the display, it appears as a sinusoidal grating with a clearly visible center and blurred edges in the periphery. In this paradigm, the horizontally moving Gabor elements had different phase shifts between the eyes (Figure 1B). Throughout the task, observers perceived the rotation of the cylinder as either clockwise, counterclockwise or ambiguous (i.e., no depth). If the left eye was delayed, observers would perceive a cylinder rotating with a counterclockwise rotation (Figure 1A). Hence, on the basis of the above literature, if the Gabor elements presented to the left eye stimulus were composed of higher spatial frequencies compared with that shown to the right eye, one would expect that the left eye would process stimuli more slowly; therefore, the observer would see a counterclockwise rotation of the cylinder. Conversely, if the right eye was delayed because the elements making up its image comprised Gabor elements of higher spatial frequency, one would expect processing associated with the right eye to be slower, thereby resulting in a clockwise percept for the rotation of the cylinder. If we also assumed that size is processed similarly to spatial frequency, in which larger sizes are processed faster, we would expect a counterclockwise rotation of the cylinder when the left eye was shown images composed of Gabor elements of smaller size compared with that shown to the other eye.

## 2. Materials and Methods 

### 2.1. Participants

Seven adults with normal vision participated in this study (mean age = 28.6 ± 4.7 years, one author, five females). This research was approved by the Ethics Review Boards of McGill University Health Center and is in line with the tenets of the Declaration of Helsinki. All subjects provided written informed consent. Two subjects were excluded because they could not perform the task accurately.

### 2.2. Apparatus

We programmed the stimuli and experiment using Matlab 2015a with the PsychToolBox extension 3.0.8 [24]. The experiment was run on an Apple MacPro computer with a Linux Mint operating system and an Nvidia GeForce GT graphics card. The dichoptic stimuli were presented on a wide 23-inch 3D-Ready LED monitor ViewSonic V3D231. The screen was gamma-corrected and had a mean luminance of 100 cd/m^2^ at a resolution of 1920 × 1080 pixels and a refresh rate of 60 Hz in an interleaved line stereo mode. Subjects performed the experiments in a dimly lit room at 90 cm viewing distance from the screen. Subjects wore polarized 3D glasses throughout the study; this reduced luminance by 60% and generated a crosstalk of 1%.

### 2.3. Stimuli and Procedure

**Figure 2:**
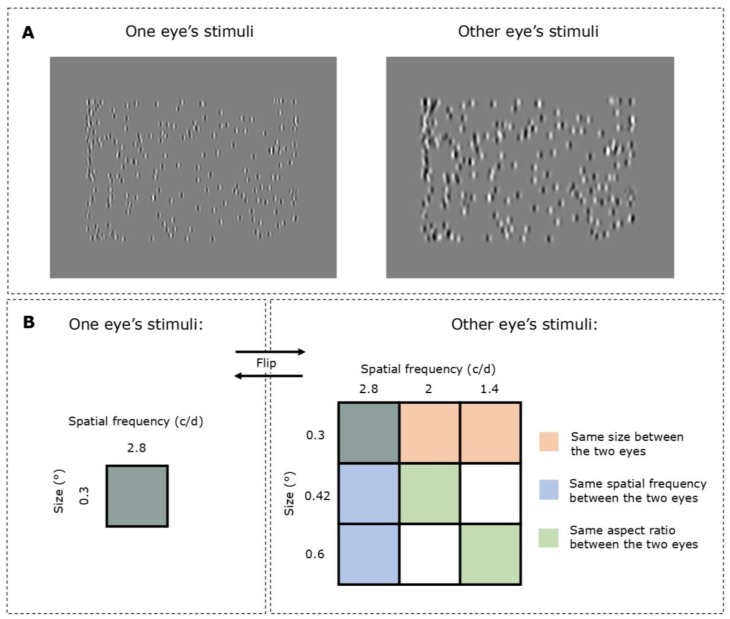
Stimuli and the experiment design. (**A**) The stimulus was presented dichoptically, with differing or the same values of either spatial frequency and/or size of the Gabor elements in each eye. In this example, the left eye is shown Gabor elements at 0.3° size (sigma parameter of the Gabor) and 2.8 c/d spatial frequency, whereas the right eye is shown Gabor elements at 0.42° size and 2.0 c/d spatial frequency. (**B**) A diagrammatic explanation of the experiment design. Subjects were shown Gabor elements that had a spatial frequency of 2.8 c/d and size of 0.3 degrees to one eye and a combination of other spatial properties to the other eye. The conditions were then reversed so that the former eye would be shown Gabor elements from the combinations of spatial frequency and size.

The stimulus consisted of two dichoptically presented arrays of Gabor elements that moved horizontally in the plane of the screen (see Figure 1). Each array subtended a 18° × 12° visual angle and was composed of 200 Gabor elements (see Figure 2A), which oscillated between the left and right of the screen with a sinusoidal angular speed of 18°/sec (degrees of phase) for a duration of 800 ms. One eye was presented with Gabor elements that had a size of 0.3° (sigma parameter of the Gabor elements) and a spatial frequency of 2.8 c/d. The other eye was presented with Gabor elements that had a size (sigma) of either 0.3°, 0.42° or 0.6° and a spatial frequency of 1.4 c/d, 2.0 c/d or 2.8 c/d (see Figure 2B). All combinations of conditions were completed in a counter-balanced fashion so that both right and left eyes were presented with all the established sizes and spatial frequencies while the other viewed a 0.3° size and 2.8 c/d spatial frequency. There was no fixation point in this task. 

**Figure 3:**
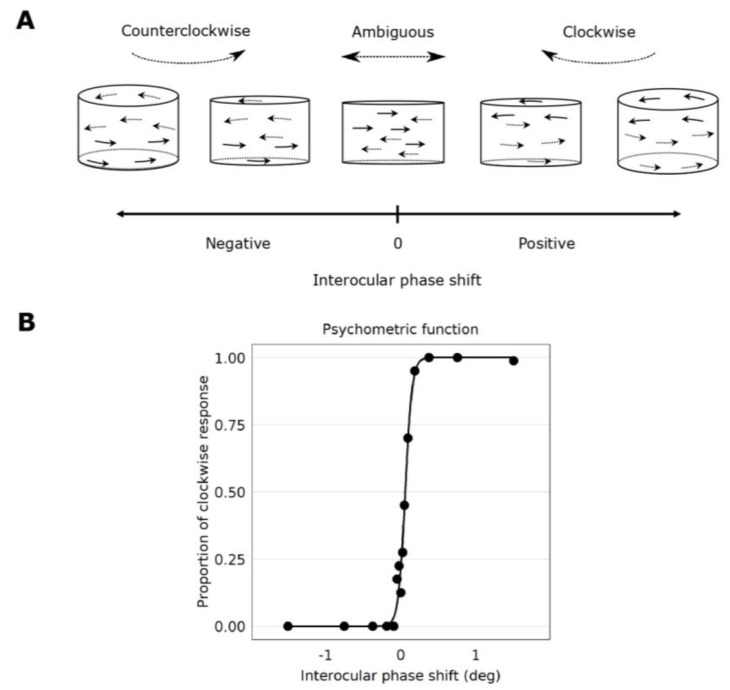
Psychometric function fits and the perceived rotation of the cylinder stimulus as a function of interocular phase shift. (**A**) When there is an interocular phase shift in the oscillation of the Gabor elements, the cylinder appears to rotate in depth. When the phase shift is negative, the rotation of the cylinder is seen counterclockwise. When the phase shift is positive, the rotation of the cylinder is seen clockwise. When the phase shift is 0°, the depth percept appears ambiguous, whereby Gabor elements are seen moving laterally in the plane of the screen. This figure has been adapted from Reynaud and Hess, 2017 [5]. (**B**) A psychometric function of the perceived rotation direction as a function of the interocular phase shift between the eyes from Subject 7. Datapoints were fitted by a logistic function. Each data point represents each interocular phase shift that was tested. The interocular phase shift for which the proportion seen clockwise is 50% marks the point of subjective equality (PSE). It is where the rotation of the cylinder is ambiguously clockwise or counterclockwise to the observer.

During the psychophysical task, an interocular phase shift between the left- and right-eye images was introduced to induce spatial disparity (see Figure 1B). As a result, the Gabor elements followed an elliptical trajectory of motion in depth. When the phase shift was negative, the cylinder appeared to rotate in a counterclockwise fashion when viewed above; if the phase shift was positive, the cylinder appeared to rotate in a clockwise direction when viewed above (see Figure 3A). When the phase shift was zero, the Gabor elements appeared to be moving laterally left and right with no depth (see Figure 3A). This point of ambiguous perception characterizes the point of subjective equality (PSE). 

Subjects reported whether they perceived the rotation of the cylinder as clockwise or counterclockwise. We used the method of constant stimuli. For each testing block, we applied different interocular phase shifts between the two eyes: −1.5°, −0.75°, −0.375°, −1875°, −0.0938°, −0.0469°, −0.0234°, 0°, 0.0234°, 0.0469°, 0.0938°, 0.1875°, 0.375°, 0.75° and 1.5° (each dot represents each interocular phase shift in Figure 3B). There were ten repetitions in each block. One condition was tested in each block, each of which was repeated twice.

### 2.4. Data Analysis

We used the cylinder task, an appearance-based procedure, from which we measured the point of subjective equality (PSE). The PSE is the interocular phase shift (in degrees) where observers perceive the cylinder (i.e., made of 200 Gabor elements) rotating in the clockwise direction 50% of the time. Psychometric functions were fitted using a logistic function forced between 0 and 1 using Matlab R2018a and the Palamedes toolbox [25]. PSEs from these fitted psychometric functions were then estimated. A negative PSE indicates a delay in the left eye and a positive one a delay in the right eye. A psychometric function of the proportion seen clockwise as a function of the interocular phase shifts is shown in Figure 3B. 

In this paper, we report PSEs as a function of either the size or spatial frequency ratio of the Gabor elements shown to the left and right eye. We conducted linear regression as in previous studies [5,26] using RStudio [27]. We performed a two-tailed Wilcoxon test to compare the slopes of PSEs as a function of frequency and size ratio. We also report the slope of the psychometric curves as a function of both the size ratios and spatial frequency of the Gabor elements shown to each eye. For the analysis of the slope of the psychometric functions, we fitted an inverted absolute value function to the slope values using Matlab R2018a. 

In the manuscript and figures, we express the size and spatial frequency ratios between the Gabor elements shown to the left and right eye in log units (in dB) using the formula:

ratio_dB_ = 20 × log10(ratio_linear_), where

ratio_linear_ = size_LE_/size_RE_ or

ratio_linear_ = spatial frequency_LE_/spatial frequency_RE_.

When the spatial frequency of the Gabor elements shown to the left eye is twice as high as of those shown to the right eye, the ratio_linear_ is 2 and ratio_dB_ 6 dB. When the former is half the latter, the ratio_linear_ is 1/2 and ratio_dB_ −6 dB. A difference of a factor of √2 in the stimulus parameters of the Gabor patches shown to each eye results in ratio_dB_ = ±3 dB.

## 3. Results

**Figure 4:**
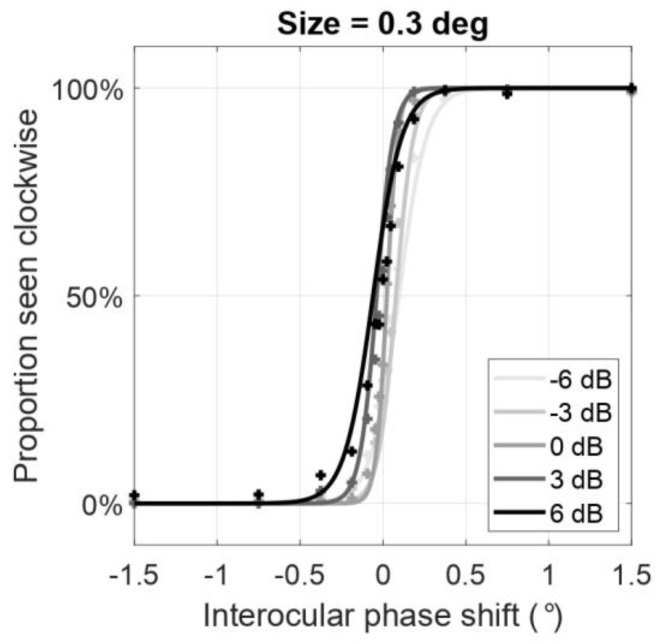
Averaged psychometric functions of the perceived rotation direction as a function of the interocular phase shift between the eyes from all observers when the size of the Gabor elements was maintained at 0.3 deg in both eyes. Each shade of grey represents different ratios between the spatial frequencies of the Gabor elements shown to left and right eye (LE/RE) from −6 dB (light grey) to 6 dB (dark grey), where 0 dB represents a linear ratio of one (same spatial frequency in the two eyes images). Datapoints were fitted by a logistic function. Interocular phase shift (indicated by *x*-axis) where the proportion seen clockwise is 50% (indicated by *y*-axis) indicates where the rotation of the cylinder is ambiguously clockwise or counterclockwise to the observer. Throughout this paper, we refer to this region as the point of subjective equality (PSE). The PSE decreases as the ratio_dB_ between the spatial frequencies in the two eyes increases.

**Figure 5:**
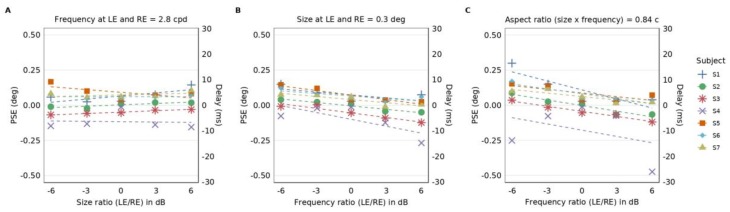
PSEs as a function of either size or spatial frequency ratio between the Gabor elements shown to the left and right eye. (**A**) Individual subjects’ PSEs (in degrees, left *y*-axis) as a function of the size ratio between the left and right eye’s Gabor elements when the frequency of the Gabor elements presented to both eyes is 2.8 c/d. Dotted lines represent linear regression for each subject. The slope from linear regression of PSEs as a function of the size ratio is not significantly different from zero (two-tailed Wilcoxon signed rank test: *p* = 0.47). Right *y*-axis represents the equivalent delay (in milliseconds). (**B**) Individual subjects’ PSEs (in degrees, left *y*-axis) as a function of the spatial frequency ratio between the Gabor elements when the size of the Gabor elements presented to both eyes is 0.3 deg. Dotted lines represent linear regressions for each subject. The slope from the linear regression as a function of the frequency ratio is significantly different from zero (two-tailed Wilcoxon signed rank test: *p* = 0.016). (**C**) Individual subjects’ PSEs (in degrees, left *y*-axis) as a function of the frequency ratio between the Gabor elements shown to the left and right eye when the aspect ratio remains at 0.84 cycles for both eyes. The aspect ratio is the product of spatial frequency and size. Dotted lines represent linear regression for each subject. As the spatial frequency of the Gabor elements shown to the left eye gets lower relative to that shown to the right eye, PSE becomes more positive. We observe that the linear regression slope is significantly different from zero (two-tailed Wilcoxon signed rank test: *p* = 0.016).

A representative subject’s psychometric functions of the proportion seen clockwise as a function of the interocular phase shifts are shown in Figure 4 when the size of the Gabor elements was maintained at 0.3 deg in both eyes. However, different spatial frequency ratios of Gabor patches were shown to the eyes (indicated by each shade of grey). Compared to the function where the spatial frequency is the same in the two eyes (0 dB, medium grey), the psychometric functions shift to the left when the frequency is decreased in the right eye (positive ratios, darker shades of grey) and shift to the right when the frequency is decreased in the left eye (negative ratios, lighter shades of grey). In other words, as the frequency ratio (LE/RE) increases, the psychometric function shifts to the left and the point of subjective equality (the point at which the curve reaches 50% performance) decreases. From these psychometric functions, we computed the PSE (deg). Then, the PSE and its linear regression were plotted as a function of either the size or spatial frequency ratio of the Gabor elements shown to the left and right eye (Figure 5).

We plotted the estimated PSEs as a function of size or frequency ratio between the Gabor elements shown to the left and right eye (see Figure 5). A negative PSE indicates a delay in the left eye and a positive PSE a delay in the right eye. Figure 5A is a plot of PSEs as a function of the ratios between the sizes of the Gabor patches shown to the left and right eye when both eyes were shown Gabor patches at the spatial frequency of 2.8 c/d. In this condition, we did not observe changes of the PSEs as a function of the size ratio between the left and right eye. We confirmed this observation by performing a linear regression between the PSE (deg) and the size ratio (dB) for all subjects [5,26]; the estimated slopes were not significantly different from zero (two-tailed Wilcoxon signed rank test: *p* = 0.47).

Figure 5B is a plot of PSEs as a function of the ratio between the spatial frequency shown to the left and right eye when both eyes were shown Gabor patches at the size of 0.3 degrees. In this condition, we observed that the PSEs decrease as a function of the spatial frequency ratio between the left and right eye. Most of the PSEs were positive for negative ratios and negative for positive ratios. We performed linear regression between the frequency ratio (dB) and the PSE (deg) and found that the estimated slopes were significantly different from zero (two-tailed Wilcoxon signed rank test: *p* = 0.016).

The relative delay becomes more evident when the magnitude of the spatial frequency gets larger, as exemplified by the trend of the PSE in Figure 5B across a range of the spatial frequency ratio. For instance, when the spatial frequency of the left eye’s Gabor elements is lower by a factor of two relative to that of the right eye, the averaged PSE is 0.064 deg, which amounts to a relative delay of 3.51 ms in the right eye.

In Figure 5C, we show a plot of PSEs for conditions where the Gabor patches had the aspect ratio of 0.84 cycles, which is the product of size (deg) and spatial frequency (c/d). The PSEs are plotted as a function of the frequency ratio between the Gabor patches shown to the left and right eye. In this plot, when the frequency ratio between the left and right eye increases, the size ratio between the eyes decreases because the aspect ratio remains the same; in other words, the spatial frequency bandwidth is kept constant. We observe a decreasing trend in PSEs as the spatial frequency in the left eye increases relative to the right eye. Here, again, we observe mostly positive PSE values for negative ratios and negative PSE values for positive ratios. The higher the spatial frequency, the slower the processing. We performed linear regression between the size ratio (dB) and PSE (deg) and found that the estimated slopes were significantly different from zero (two-tailed Wilcoxon signed rank test: *p* = 0.016).

Figure 5 demonstrates that the frequency differences between the Gabor elements shown to the left and right eye, rather than the bandwidth or size, affects PSE. To assess whether the influences on the PSEs by the frequency and size differences have a common mechanism, we assessed the correlation between regressions of PSEs as a function of the ratio of size (from Figure 5A) or spatial frequency (from Figure 5B). We found no significant correlation between the regressions (R-squared = 0.16, *p* = 0.37). The lack of correlation is in line with our findings that differing spatial frequencies but not sizes influenced the PSEs.

In short, when size is disambiguated from spatial bandwidth, the PSE is affected by spatial frequency of image features not by their size. (see Figure 5). The influence on the PSEs by the frequency and size differences do not seem to have a common mechanism, as demonstrated by a weak correlation.

**Figure 6:**
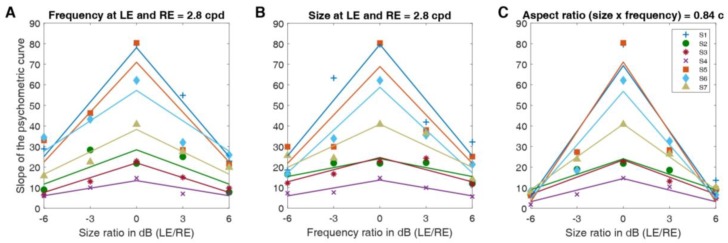
Slopes of the psychometric curve as a function of either size or spatial frequency ratio between the Gabor elements shown to the left and right eye. (**A**) Individual subjects’ slopes of the psychometric curve (*y*-axis), as a function of the size ratio between the left and right eye’s Gabor elements when the frequency of the Gabor elements presented to both eyes is 2.8 c/d. Fitted lines represent fits with an inverted absolute value function for each subject. (**B**) Individual subjects’ slopes of the psychometric curve (*y*-axis) as a function of the spatial frequency ratio between the Gabor elements when the size of the Gabor elements presented to both eyes is 0.3 deg. Fitted lines represent fits with an inverted absolute value function for each subject. (**C**) Individual subjects’ slopes of the psychometric curve (*y*-axis) as a function of the frequency ratio between the Gabor elements shown to the left and right eye when the aspect ratio remains at 0.84 cycles for both eyes. Aspect ratio is the product of spatial frequency and size. Fitted lines represent fits with an inverted absolute value function for each subject.

By computing the slope of the psychometric function as an indicator of the task difficulty, we assessed the task difficulty for each condition. The steeper the psychometric slope, the more accurate the performance of participants. We had hypothesized that when the left and right eyes viewed Gabor patches with different spatial frequencies and sizes, the task difficulty would increase. Figure 6 represents the slopes of the psychometric curves for all subjects for differing sizes and frequencies (Figure 6A,B respectively), as well as the same aspect ratio (Figure 6C), as a function of the size or spatial frequency ratio between the Gabor elements shown to the left and right eye. In all conditions, and for all participants, we observed that the slopes were steepest when the size and frequency ratios in dB were zero (i.e., with the same values in both parameters between the eyes), and lowest when the ratios were large (large differences in both parameters between the eyes). This observation indicates that the task gets harder when the difference between the images presented to the two eyes is more pronounced. In other words, our findings demonstrate that the slope of the psychometric curves for individual subjects decrease similarly with differing sizes (see Figure 6A) and frequencies (see Figure 6B). To assess how differing sizes and frequencies influence the slope of the psychometric curves, we fitted these slopes with a negative absolute value function (‘V’ shaped fits are positive absolute value functions). 

**Figure 7:**
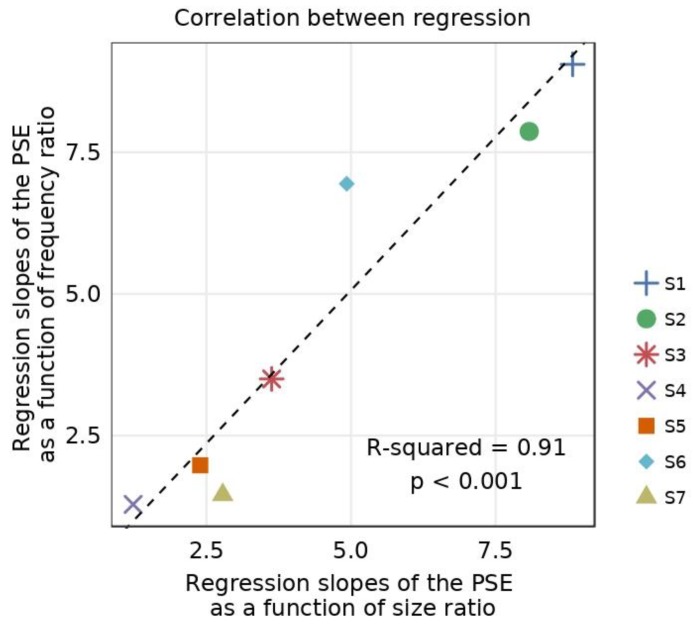
Correlation between the amplitude coefficients of the inverted absolute fit for differing size and frequency ratios. Regression slopes of the PSE for differing frequencies (*y*-axis) as a function of the regression slopes of the PSE for differing sizes (*x*-axis). The slopes were obtained from linear regression between PSEs and the ratio of size or spatial frequency in Figure 6A,B. The dotted line represents correlation between the two linear regressions.

In Figure 7, we report the correlation between the amplitude coefficients of the absolute fits of the slope as a function of the ratio of size (from Figure 6A) or spatial frequency (from Figure 6B). We found a significant correlation between the absolute fits (R-squared = 0.91, *p* < 0.001). In short, the slope of the psychometric function decreases similarly when either the spatial frequency or the size of the Gabor elements differs between the eyes (see Figure 6).

## 4. Discussion

When information entering one eye gets delayed, the observer perceives an elliptically rotating pendulum even though its actual trajectory is in the fronto-parallel plane. In this study, we measured interocular (i.e., between the eyes) delay using a paradigm with a cylinder stimulus composed of 200 laterally moving Gabor elements. Depending on the duration of interocular delay, the cylinder’s rotation can be perceived to be clockwise or ambiguous or counterclockwise. We manipulated two stimulus parameters—spatial frequency and the size of the Gabor elements defining the cylinder— and varied their interocular values. For instance, one eye would be shown Gabor patches of a higher spatial frequency or smaller size than that shown to the other eye. These parameters are pertinent to the spatial property of visual stimuli. Previous neurophysiological studies have reported that these parameters modulate the speed of visual processing from the lateral geniculate nucleus [28] to the primary visual cortex [29,30,31,32,33] and extra-striate areas [30,34]. These studies collectively show that visual information represented by low spatial frequencies is processed faster than that represented by high spatial frequencies within the visual pathway. 

Our findings support the notion that spatial frequency influences the speed of visual processing. They are in line with previous studies that report that low spatial frequency information is processed faster than high spatial frequency information [13,15,17]. For example, we observed interocular delay when the spatial frequency of the Gabor elements differs between the eyes. A higher spatial frequency in one eye relative to that in the other eye resulted in a perception (with a resultant PSE shift) consistent with a delay in the processing of information in the that eye. In other words, changing the spatial frequencies between the eyes induces a Pulfrich effect in a predictable direction. However, interocular stimulus size differences did not influence the Pulfrich effect nor, by implication, the dynamics of visual processing. 

Our results show that human observers can binocularly fuse visual stimuli that differ in spatial frequency or size between the eyes. When the sizes (bandwidths) and spatial frequencies of the stimuli differ between the eyes, these stimuli do not result in either perceptual suppression or diplopia and the expected relationship between disparity and psychometric performance is obtained (slope > 0). Furthermore, we can also infer the range of interocular differences in spatial frequency where fusion breaks down by pinpointing where on the *x*-axis, be it spatial frequency or size ratio, the slope of the psychometric curve falls to zero in Figure 6. The slope falls to zero when the spatial frequency and size ratios of the Gabor elements are approximately ±10 dB for most subjects. The visual system is able to fuse these visual stimuli when the interocular size or spatial frequency difference is less than a factor of five.

There have been other reports of fusion for stimuli that differ in spatial frequency, involving rivalry and tilt. First, when physically different stimuli are presented to each eye, perception of one stimulus can dominate over another in an alternating fashion; this phenomenon is known as binocular rivalry [35]. However, when the physically different stimuli shown to the two eyes represent the same object within an identical location, the stimuli can nevertheless be binocularly fused [36]. Previous studies have reported fusion when both eyes viewed rivalrous dichoptic stimuli [37,38,39] that were presented for as short as 150 ms [40]. This is not directly relevant to our stimuli, as they were presented for 800 milliseconds and the perception of rivalry did not occur. In addition, there have been reports that a perception of tilt, which is depth about the vertical axis, has been observed with stimuli of different interocular spatial frequencies across a wide range (0.5 to 15 c/d) [41,42]. However, this is not relevant to the present results. The tilt is only observed with extended grating stimuli that have a larger number of cycles (more than eight cycles) [41,42,43] and hence a narrower orientation bandwidth. This does not apply to our stimuli, which were composed of local micropatterns with a broad orientational bandwidth and for which only horizontal disparity was varied. 

Our findings show that the presentation of stimuli with an interocular spatial frequency difference induces a Pulfrich effect. This could be because of an imbalance in the processing speed of high (i.e., slower) and low (i.e., faster) spatial frequencies prior to binocular combination. The relative delay can increase as the spatial frequency difference increases (see Figure 5B). When we present information at a factor of two lower spatial frequency to the left eye relative to that shown to the right eye, the resultant Pulfrich effect is consistent with a relative delay of 3.51 ms. Reynaud and Hess (2017) found a comparable delay of 4 ms in observers when viewed with stimuli containing 60% interocular contrast difference [5]. Therefore, an interocular spatial frequency difference of a factor of two and an interocular contrast difference of 60% induce interocular delays of similar magnitudes. These findings collectively suggest that properties of a visual target that influences the speed of visual processing prior to binocular combination can induce the Pulfrich effect. 

Physiological studies indicate that broadband stimuli are processed most quickly [44,45]. These results indicate that our observations could be due to an imbalance between the durations for processing low and high spatial frequencies. A recent study demonstrates the effect of differential blur (i.e., removing high spatial frequency content from an image seen by one eye) in driving the Pulfrich effect [46]. The authors found that blurring the image in one eye induces the Pulfrich effect by increasing the speed of visual processing in said eye compared to a broadband stimulus presented to the other eye. Therefore, it seems that the absence of high spatial frequency content, rather than the presence of low spatial frequency content, speeds up visual processing [46]. 

We had expected subjects to show a relative delay in the eye viewing stimuli of a higher spatial frequency. However, some individuals exhibited a relative delay in the eye that viewed Gabor elements of a lower spatial frequency (Subjects 1 and 6, see Figure 5B). They had unilateral delays (i.e., delay exclusively in one eye) in all conditions. Nevertheless, the delay turned out to be longer in the eye viewing Gabor elements of higher spatial frequency and followed a common trend of getting more negative as the spatial frequency ratio increased (Figure 5B). We attributed the unilateral delay in all conditions to an innate interocular delay in these exceptional subjects [5,47,48,49,50,51,52]. If some subjects already had a large innate interocular delay, this delay would nonetheless persist even when the eye was shown images with a lower spatial frequency content.

## 5. Conclusions

In summary, we have shown that physically different visual features (in spatial frequency or size) presented to different eyes can be fused if they are part of the same visual object. Furthermore, presentation of image features with different spatial frequencies to different eyes influences the Pulfrich effect, a result that is consistent with findings that show that the low spatial frequency content is processed faster than the high spatial frequency content. However, presentation of stimuli with different sizes or spatial bandwidth to different eyes does not induce the Pulfrich effect.
